# An unusual presentation of giant sebaceous cyst over the back: A case report

**DOI:** 10.1002/ccr3.8714

**Published:** 2024-03-29

**Authors:** Neel Doshi, Pratik Gond, Tanisha Prasad, Abhigan Babu Shrestha

**Affiliations:** ^1^ Pravara Institute of Medical Sciences Ahmednagar India; ^2^ University of Medicine and Health Science, Royal College of Surgeons in Ireland Dublin Ireland; ^3^ M Abdur Rahim Medical College Dinajpur Bangladesh

**Keywords:** cancer, dermatology, epidermal cysts, sebaceous cyts

## Abstract

Sebaceous cysts are a prevalent form of benign cutaneous cysts that often manifest as epidermal cysts when their size exceeds 5 cm in diameter. Despite their infrequent occurrence, cases of massive epidermal cysts measuring 5 cm or larger have been documented. Malignant transformations within epidermal cysts are exceedingly rare but not impossible. Although their development into malignancies is a rare event, it is essential to acknowledge the potential for benign epidermal cysts to undergo such transformation. In clinical terminology, these cysts predominantly affect the facial, cervical, and dorsal regions. They usually remain asymptomatic, with their formation attributed to the sequestration of epithelial remnants during embryonic fusion or trauma. In this report, we present a remarkable case of a colossal epidermal cyst found on the upper back of a 75‐year‐old male individual, highlighting the uncommon nature of such occurrences within the medical domain. This case adds to the literature by presenting a rare case report highlighting the clinical presentation, probable consequences, and surgical therapy of larger epidermal cysts.

## 1 INTRODUCTION

Sebaceous cysts, often known as epidermoid cysts, are keratin‐containing unilocular retention cysts. It frequently appears on the head, neck, scalp, scrotum, earlobe, and breast, and can range in size from a few millimeters to less than a few centimeters. When an epidermal cyst exceeds more than 5 cm in diameter, it is considered a giant cyst. The development of cancer is more likely in giant epidermal cysts, which are uncommon.^1^

Conventional sebaceous cysts are typically small, slowly expanding, nonsensitive lesions in dome shape. Unless it becomes infected or enlarges to the point where it damages nearby anatomical structures, an epidermal cyst is typically asymptomatic. Authors have previously described enormous epidermal cysts with diameters greater than 5 cm.^2^, ^3^

A well‐developed granular cell layer lines epidermoid cysts, which are also lined by stratified squamous epithelium. On rare occasions, the cyst wall may also contain pseudostratified ciliated columnar epithelium. The cyst wall may have calcification of a dystrophic type. The preferred course of action is excision. Studying the occurrence of enormous sebaceous cysts was the goal.^4^

Epidermal inclusion cysts, ganglion cysts, neurogenic tumors, myxoid tumors, nodular fasciitis, and dermatofibrosarcoma protuberans are among the possible diagnoses. The reported incidence of malignant degeneration to squamous cell carcinoma is 2.2%.^5^

The present study reported a case of a giant sebaceous cyst over the posterior upper back in a 75‐year‐old male.

## 2 CASE HISTORY

A 75‐year‐old male patient came to the outpatient surgery department at Pravara Rural Hospital in Loni complaining of a 5‐year‐old swelling that had been gradually getting worse. The cyst was present over the left upper back and shoulder region. The history of presenting complaint revealed that the swelling was approximately 1 cm in size when he first noticed it a few months ago but gradually grew in size until it reached a large one. The patient also described experiencing discharge from the swelling for the last 6 months.

### 2.1 Methods

**FIGURE 1:**
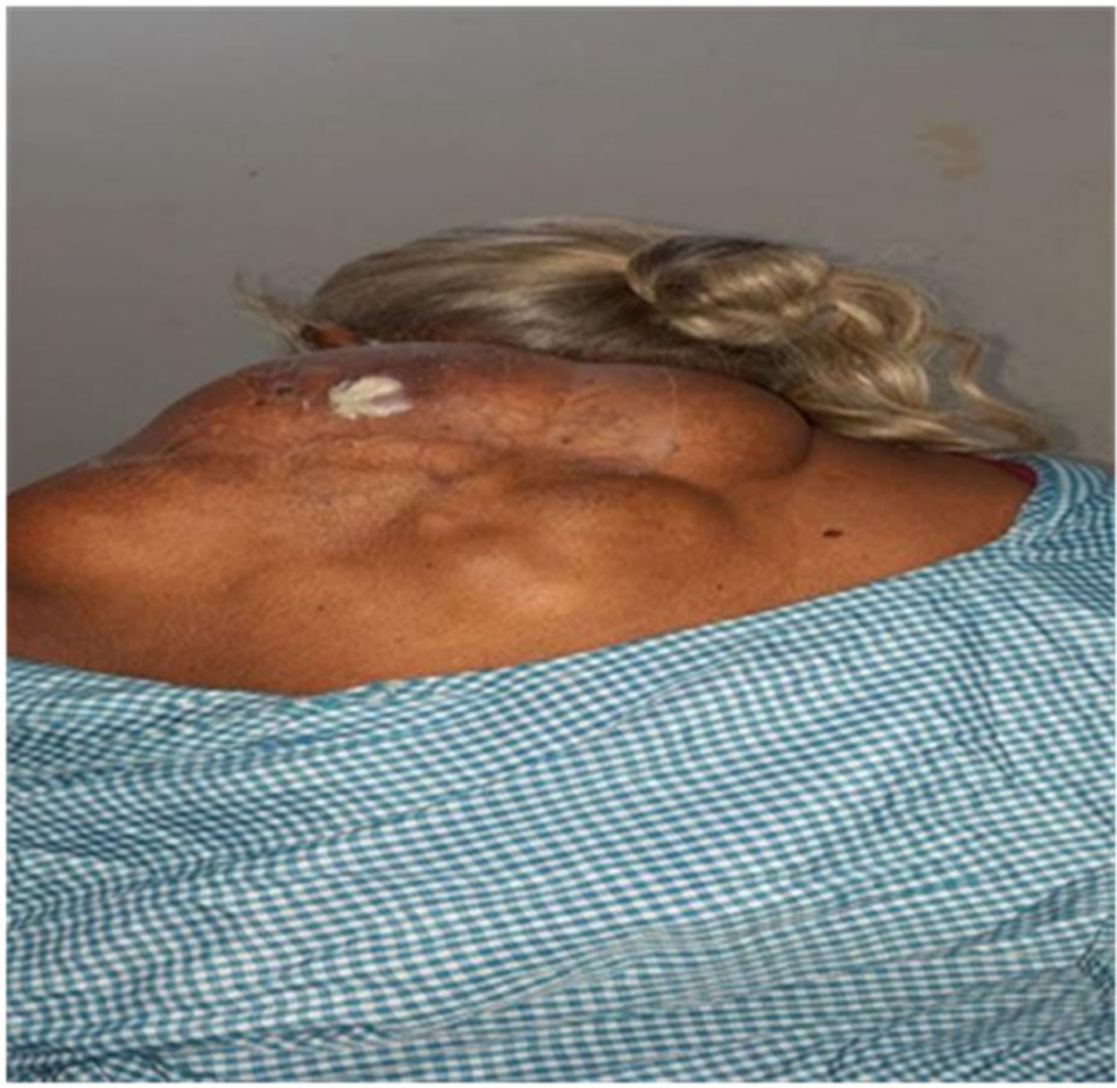
Multi‐lobulated soft cystic swelling measuring 20 × 17 × 10 cm swelling over the left upper back and shoulder.

An extensive multi‐lobulated soft cystic swelling measuring 20 cm by 17 cm by 10 cm was found during the clinical examination of the swelling over the left upper back and shoulder (Figure 1). There was a cream‐colored discharge that had the consistency of butter and was oozing from the swollen punctum.

### 2.2 Investigations

**FIGURE 2:**
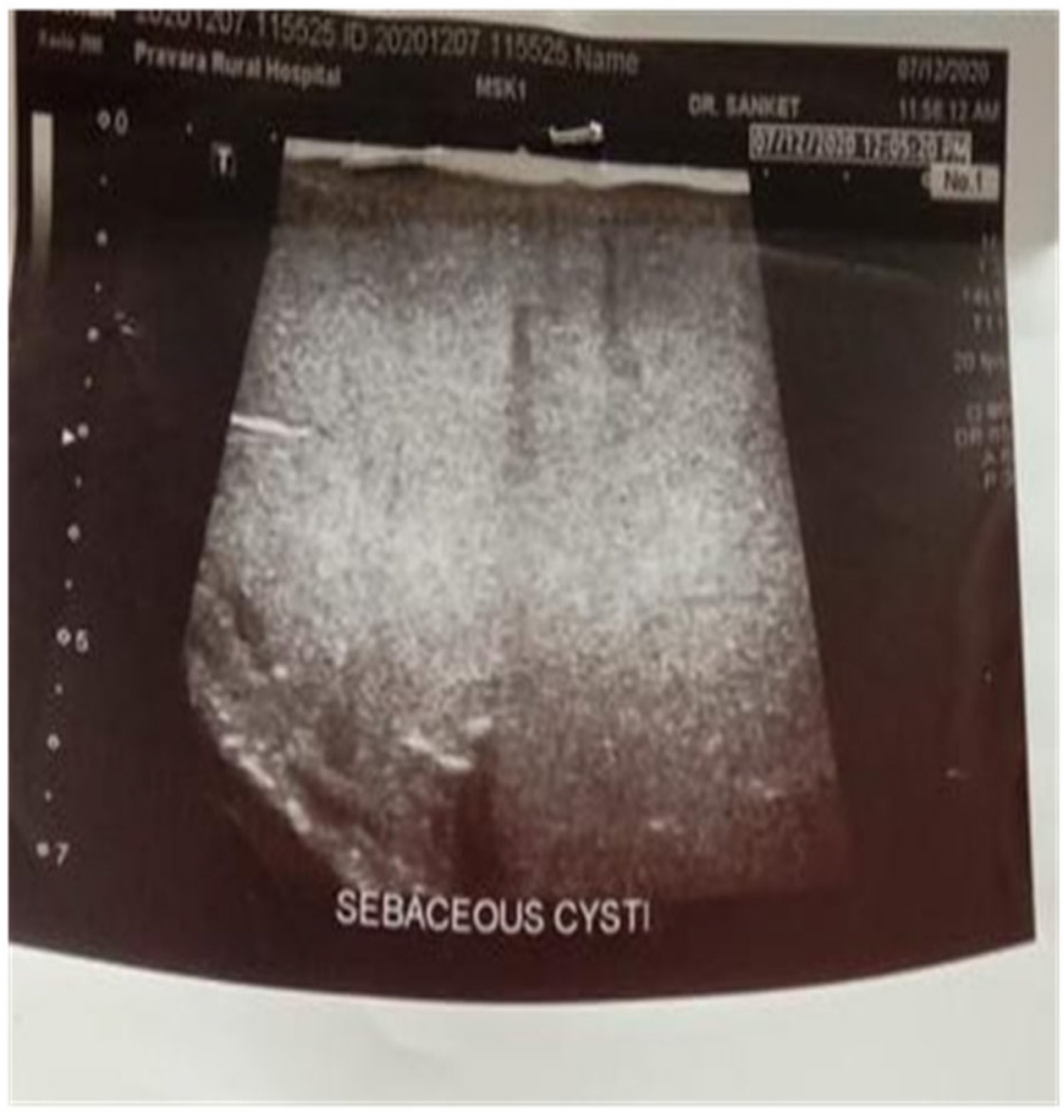
Ultrasound results showed a large, well‐defined, lobulated, and heterogeneous hypoechoic lesion with numerous small calcifications.

On ultrasound of the lesion, the results showed a large, well‐defined, lobulated, heterogeneous hypoechoic lesion with numerous small calcifications on the left upper back region (Figure 2). Doppler study of the cyst revealed no internal vascularity or a solid component within the swelling's underlying etiology. In the subcutaneous plane of the cervical region and upper back region on the left side, there was a multi‐lobulated area of altered signal intensity that measured roughly 8.5, 10.4, 14.8 cm (AP, TR, CC), appeared hyperintense on T2W and STIR images, and appeared hyperintense on T1W images. DWI showed diffusion restrictions in these regions. Indistinct fat planes with the trapezius are visible anteriorly. The trapezius was displaced laterally, anteriorly, and posteriorly over the skin as a result of this lesion. A thin peripheral enhancement was discovered during the post‐contrast study. Fat in the vicinity seems normal. Findings that could point to sebaceous cysts.

## 3 TREATMENT

**FIGURE 3:**
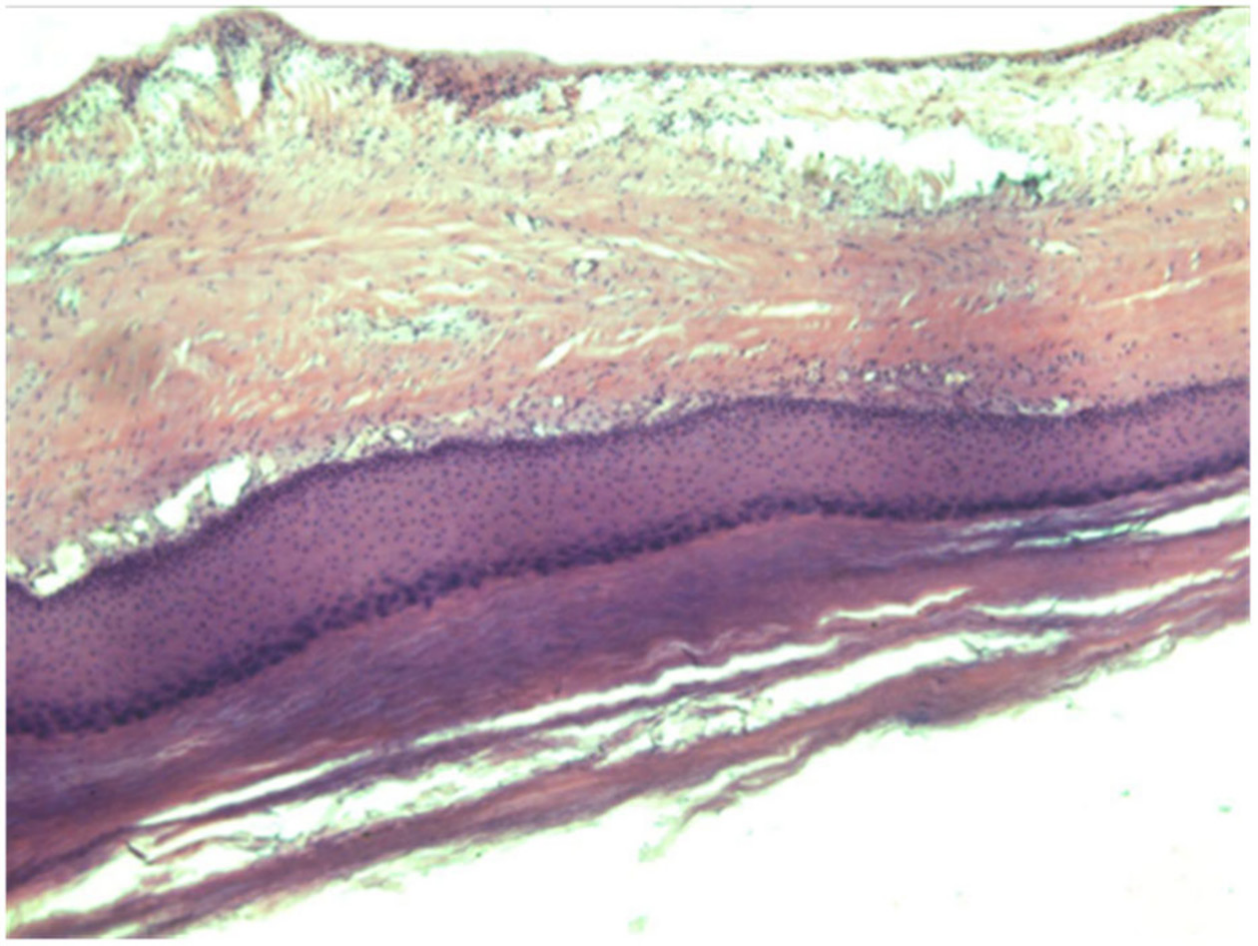
Histopathology revealed a cyst with a stratified squamous epithelium lining that contained keratin.

**FIGURE 4:**
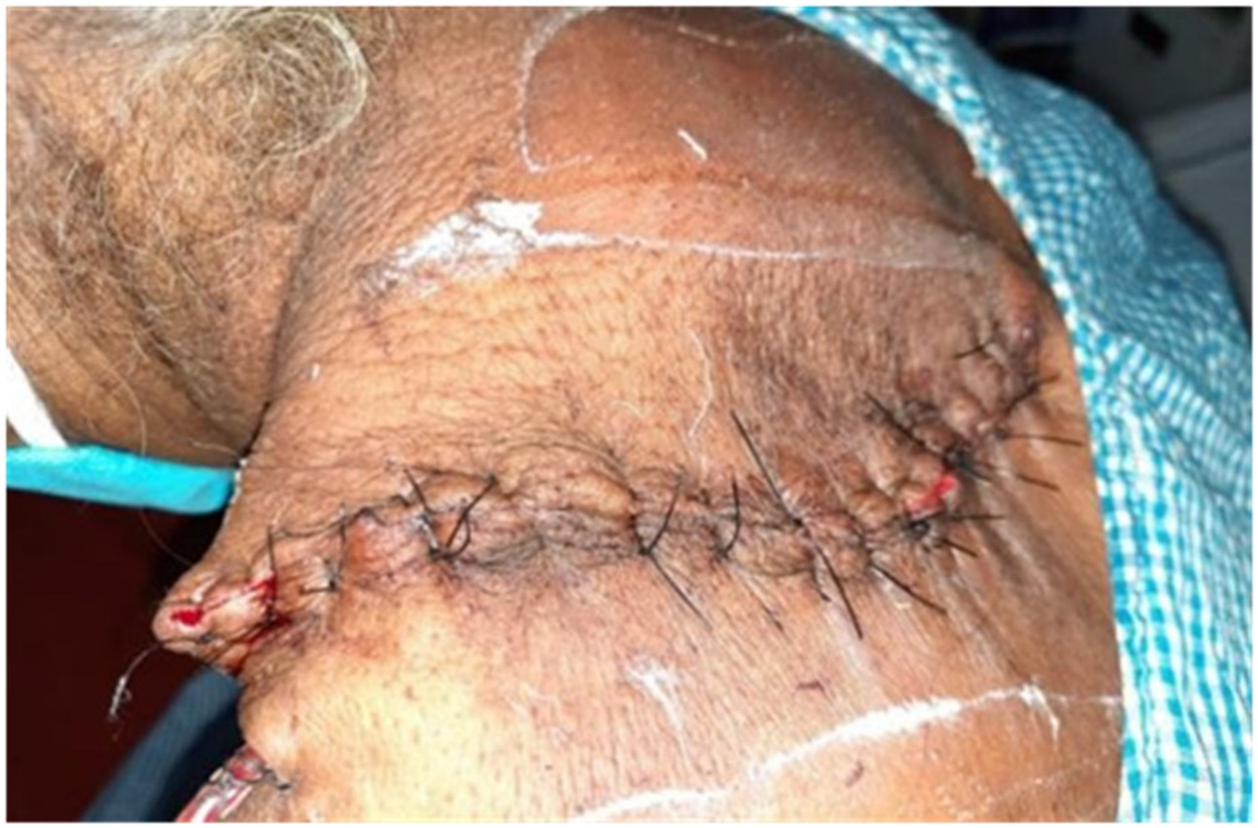
Romovac drain was inserted and the skin was closed with Ethilon 2‐0 mattress sutures.

The specimen's histopathology revealed a cyst with a stratified squamous epithelium lining that contained keratin (Figure 3). The histopathology result ruled out any malignant changes. The patient was intubated under general anesthesia an oblique elliptical incision was made around the swelling. Incision deepened up to the cyst wall and a total skin flap was excised from the cyst and underlined muscle and hemostasis was achieved. Romovac drain was inserted and the skin was closed with Ethilon 2‐0 mattress sutures (Figure 4). After the cystic mass has been removed, excess skin is removed to promote primary closure and aesthetic improvement. A day after surgery, the patient was released, and on the 3rd day of follow‐up, there were no complaints. On the 10th postoperative day, the stitches were removed after the wound healed without any complications.

## 4 CONCLUSION AND RESULTS

At the follow‐up examination at regular intervals of 15 days, the authors found no local recurrence of the lesion.

Despite being a benign, slow‐growing tumor, the epidermal cyst can make diagnosis challenging. In this case, the authors show that a giant epidermal cyst can grow for a long time and have negative effects on the surrounding structures as well as serious cosmetic issues that may call for mental health counseling. Therefore, for patients with a giant epidermal cyst, early surgical excision is advised.

This case, thus highlights the rarity of large epidermal cysts, which often measure more than 5 cm in diameter. This case report contributes to the understanding of the clinical manifestations of such cysts. It also emphasizes the possibility of benign epidermal cysts' malignant transformation, an uncommon but serious risk. Furthermore, the full clinical description and surgical management provide important assistance for other healthcare practitioners dealing with comparable instances. Overall, this abstract adds to the existing literature by giving a unique case study focusing on the clinical presentation, probable consequences, and surgical options for large epidermal cysts.

## 5 DISCUSSION

Epidermoid cysts are benign, slow‐growing, high, round, firm, subcutaneous, or intradermal cysts that typically grow 1–5 cm in diameter and are usually asymptomatic. It is noteworthy that an epidermoid cyst with a diameter ≥5 cm is rare.^6^

Rarely do giant sebaceous cysts or epidermoid cysts appear in surgical practices. These are rare before puberty but can happen at any age. Young adult males are the most common age of presentation. The face, trunk, neck, scalp, scrotum, ear lobe, and breast are the most frequent sites of occurrence, but occurrence at an unusual site is cause for concern.^4^

Epidermoid cysts with increased sizes are more brittle and prone to secondary infection. Another crucial point is that the patient may experience depression and anxiety due to the lesion's aesthetic appearance if these enormous cysts appear in the head or neck due to the high visibility of these areas.^7^

A massive epidermal cyst, which is uncommon in surgical practice. According to pathology, the cornified epithelium‐lined epidermal cyst has a distinctive granular layer, and lamellated keratin, and is not calcified. There are three different types of lesions: (1) congenital sequestration of surface ectoderm, (2) pilosebaceous unit occlusion, and (3) implantation of epidermal cells into the dermis as a result of surgical intervention and puncturing injury.^8^

In our case, there were no clinical indications of infection. As there was no nodal involvement and the lesion was benign, neoplastic conditions were ruled out. Lipomas and salivary vascular lesions are additional conditions to consider, but with the aid of a thorough physical examination and additional tests like ultrasound, and MRI we were able to rule out these other pathological conditions. Importantly, there are reports of malignant changes in epidermoid cysts in the literature. In our case, the pathology‐analyzed specimen revealed no malignancy.

Although epidermoid cysts have a benign clinical course, there have been a few isolated reports of basal cell carcinoma, squamous cell carcinoma, epithelioid carcinoma, and other malignancies being linked to these cysts. In order to ensure complete removal and prevent recurrence, complete surgical removal is the preferred course of treatment. To get good aesthetic results for giant epidermal cysts, redundant skin must be removed.^8^

A sebaceous cyst is completely removed along with its capsule as part of the treatment. In one of the few cases to date presented in the literature, the authors describe a rare instance of a massive epidermal cyst that covered the back and required total excision. FNAC typically makes a diagnosis. In order to establish the diagnosis in unusual locations, MRI is a helpful adjunct. Simple excision is the preferred procedure in uncomplicated cases, but regional perforator island flap reconstruction has been used in patients with large epidermal cysts and underlying medical conditions. The outcome of these flaps is determined by the underlying illness and any coexisting diseases.^4^, ^9^

## AUTHOR CONTRIBUTIONS

**Neel Doshi:** Supervision; writing – original draft; writing – review and editing. **Pratik Gond:** Visualization; writing – original draft; writing – review and editing. **Tanisha Prasad:** Writing – original draft; writing – review and editing. **Abhigan Babu Shrestha:** Writing – original draft; writing – review and editing.

## FUNDING INFORMATION

No funding was received for this study.

## CONFLICT OF INTEREST STATEMENT

The authors declare no conflicts of interest.

## ETHICS STATEMENT

Not applicable.

## CONSENT

Written informed consent was obtained from the patient to publish this report in accordance with the journal's patient consent policy.

## Data Availability

Data will be available on request to the corresponding author.
